# Scale-up of collaborative TB/HIV activities in Guyana

**DOI:** 10.26633/RPSP.2017.6

**Published:** 2017-02-08

**Authors:** Brian J Baker, Brandy Peterson, Jeetendra Mohanlall, Shanti Singh, Collene Hicks, Ruth Jacobs, Ruth Ramos, Barbara Allen, Eric Pevzner

**Affiliations:** 1 U.S. Centers for Disease Control and Prevention U.S. Centers for Disease Control and Prevention AtlantaGeorgia United States of America U.S. Centers for Disease Control and Prevention, Atlanta, Georgia, United States of America.; 2 Ministry of Health, National TB Programme Ministry of Health, National TB Programme Georgetown Guyana Ministry of Health, National TB Programme, Georgetown, Guyana.; 3 Ministry of Health, National AIDS Programme Secretariat Ministry of Health, National AIDS Programme Secretariat Georgetown Guyana Ministry of Health, National AIDS Programme Secretariat, Georgetown, Guyana.; 4 Division of Global HIV/AIDS, U.S. Centers for Disease Control and Prevention Division of Global HIV/AIDS, U.S. Centers for Disease Control and Prevention Georgetown Guyana Division of Global HIV/AIDS, U.S. Centers for Disease Control and Prevention, Georgetown, Guyana.; 5 Ministry of Health, National Care and Treatment Centre Ministry of Health, National Care and Treatment Centre Georgetown Guyana Ministry of Health, National Care and Treatment Centre, Georgetown, Guyana.

**Keywords:** Tuberculosis, HIV, latent tuberculosis, isoniazid, Guyana, Caribbean region, Tuberculosis, VIH, tuberculosis latente, isoniazida, Guyana, Región del Caribe

## Abstract

**Objective.:**

To assess scale-up of recommended tuberculosis (TB)/HIV activities in Guyana and to identify specific strategies for further expansion.

**Methods.:**

Medical records and clinic registers were reviewed at nine TB clinics and 10 HIV clinics. At TB clinics, data were collected on HIV testing and antiretroviral therapy (ART) for patients with TB/HIV; at HIV clinics, data were collected on intensified case finding (ICF), tuberculin skin test (TST) results, and provision of isoniazid preventive therapy (IPT).

**Results.:**

At TB clinics, among 461 patients newly diagnosed with TB, 419 (90.9%) had a known HIV status and 121 (28.9%) were HIV-infected. Among the 63 patients with TB/HIV, 33 (52.4%) received ART. Among the 45 patients with TB/HIV for whom dates of HIV diagnosis were available, 38 (84.4%) individuals knew their HIV status prior to TB diagnosis. At HIV clinics, among 127 patients eligible to receive a TST, 87 (68.5%) received a TST, 66 (75.9%) had a TST result, seven (10.6%) had a newly positive result, two had a previously positive result, and six of nine patients with positive results (66.7%) received IPT. ICF could not be assessed because of incomplete or discrepant documentation.

**Conclusions.:**

An in-depth evaluation of TB/HIV activities successfully identified areas of success and remaining challenges. At TB clinics, HIV testing rates are high; further scale-up of ART for persons with TB/HIV is needed. At HIV clinics, use of TST to focus IPT is a feasible and efficient strategy; improving rates of annual TST screening will allow for further expansion of IPT.

## RESUMEN

**Objetivo.:**

Evaluar la ampliación de las actividades recomendadas contra la tuberculosis (TB) y la infección por el VIH en Guyana y definir estrategias específicas para nuevas expansiones.

**Métodos.:**

Se examinaron los expedientes médicos y registros clínicos de nueve consultorios de atención de la TB y diez consultorios de atención de la infección por el VIH. En los consultorios de atención de la tuberculosis, se recopilaron datos sobre las pruebas de detección del VIH y el tratamiento antirretroviral (TAR) para pacientes con TB e infección por el VIH; en los consultorios de atención de la infección por VIH, se recopilaron datos a partir de una búsqueda intensiva de casos, los resultados de la prueba de la tuberculina y la provisión de tratamiento preventivo con isoniazida.

**Resultados.:**

En los consultorios de atención de la tuberculosis, de 461 pacientes recién diagnosticados con tuberculosis, 419 (90,9%) conocían su estado con respecto a la infección por el VIH y 121 (28,9%) estaban infectados por el virus. De los 63 pacientes con TB e infección por el VIH, 33 (52,4%) recibieron TAR. De los 45 pacientes con TB e infección por el VIH cuya fecha de diagnóstico de la infección por el VIH se conocía, 38 (84,4%) supieron de su estado con respecto a la infección por el VIH antes de recibir el diagnóstico de la tuberculosis. En los consultorios de atención de la infección por el VIH, de 127 pacientes que reunían los requisitos para la prueba de la tuberculina, 87 (68,5%) recibieron la prueba, 66 (75,9%) tuvieron un resultado, siete (10,6%) tuvieron un resultado positivo nuevo, dos habían tenido un resultado positivo anteriormente, y seis de nueve pacientes con resultados positivos (66,7%) recibieron tratamiento preventivo con isoniazida. No pudo evaluarse la búsqueda intensiva de casos debido a que la documentación estaba incompleta o era discrepante.

**Conclusiones.:**

Una evaluación exhaustiva de las actividades contra la TB y la infección por el VIH permitió determinar las áreas donde se había tenido éxito y los retos pendientes. En los consultorios de atención de la tuberculosis, las tasas de realización de pruebas de detección del VIH son elevadas; se necesita ampliar el alcance del TAR para llegar a las personas con TB e infección por el VIH. En los consultorios de atención de la infección por el VIH, el uso de la prueba de la tuberculina para focalizar la búsqueda intensiva de casos es una estrategia factible y eficaz; el mejoramiento de las tasas de tamizaje anual con la prueba de la tuberculina permitirá ampliar la búsqueda intensiva de casos.

Worldwide, tuberculosis (TB) remains the leading cause of death among persons living with HIV (PLHIV) (1). Guyana has been affected by the dual epidemics of TB and HIV, with an estimated adult HIV prevalence of 1.4% (2) and an estimated TB incidence of 109 per 100 000 persons (3); an estimated 35.1% of patients with TB disease are HIV-infected (4).

To reduce TB-associated morbidity and mortality in PLHIV, the World Health Organization (WHO) recommends early initiation of antiretroviral therapy (ART) for PLHIV and scale-up of the “Three I’s”: intensified case finding (ICF), isoniazid preventive therapy (IPT), and TB infection control (IC) (5). ICF refers to the use of a standardized clinical algorithm to screen PLHIV for TB disease; PLHIV with a negative symptom assessment may then become eligible for treatment with IPT. In addition, ART and co-trimoxazole preventive therapy (CPT) are recommended for all patients with TB disease and HIV infection (TB/HIV).

Since 2000, the Guyana Ministry of Health has been scaling-up testing for HIV among patients with TB, and in 2005, TB clinics in Guyana began providing CPT to patients with TB/HIV. In 2006, an international team assessed the extent to which recommended TB/HIV practices had been implemented at TB clinics in Guyana (4). The investigators found that 76.7% of patients with TB had a known HIV status, and among patients with TB/HIV, 79.6% received CPT and 33.3% received ART.

Since 2005, several TB clinics in Guyana have begun to provide ART onsite for the duration of anti-TB treatment and to refer patients to HIV clinics for subsequent care. In addition, Guyana’s national HIV guidelines have recommended ICF, an annual tuberculin skin test (TST) for eligible PLHIV, and provision of IPT (six months of daily isoniazid) for PLHIV with TST results ≥ 5 mm and no evidence of TB disease (6).

The objectives of this evaluation were 1) to assess scale-up of recommended TB/HIV activities and 2) to identify specific strategies for further expansion of TB/HIV activities.

## MATERIALS AND METHODS

### Study design

This is a retrospective study of health care services delivered during July–December 2010 at TB and HIV clinics in Guyana. Surveys were administered to staff and patients from TB and HIV clinics to assess awareness of and adherence to TB/HIV policies and practices.

### TB clinics

The vast majority of the Guyanese population lives in Georgetown (the capital city) and other locations along the Caribbean coast, and the interior of the country is less densely populated. Nine TB clinics were selected (including the six clinics from the 2006 assessment (4) and three additional clinics), which together register more than 90% of all persons with TB in Guyana. The three additional clinics were chosen to include more patients living outside of Georgetown. At each clinic, data were abstracted from TB registers for all persons listed for anti-TB treatment during July–December 2010. As TB registers do not contain all relevant data regarding care for patients with TB/ HIV, individual medical records were reviewed for a subset of patients: all persons with TB/HIV who received care during September–December 2010. All individual medical records selected were available for review. The patient cohort enrolled in those clinics received care in 2010 when Guyana’s national guidelines recommended ART within eight weeks of initiating anti-TB treatment for PLHIV with a CD4 count ≤ 350 (6).

### Register review and medical record abstraction at TB clinics

From TB registers, data were collected on HIV status; for patients with TB/HIV data were also collected from medical records for CD4 count, receipt of CPT and ART, and anti-TB treatment outcomes. Anti-TB treatment outcomes were categorized as “favorable” (cure or treatment complete); “poor” (death from any cause, treatment failure, or default); or “unknown” (unknown, transferred, or still on treatment) following definitions from national guidelines (7).

### HIV clinics

A convenience sample was selected of 10 HIV clinics providing clinical services for more than 80% of all persons accessing HIV care in Guyana in 2010. Clinics could be selected if they were located in close proximity (< 1 km) to the nine selected TB clinics, as these sites cared for the same patients with TB/HIV who attended the TB clinics already selected. The selected clinics included both public and private practice environments. HIV registers were examined for patients who had visited a clinic at least once during July–December 2010. All patients listed in these registers were then eligible for inclusion into the study. Two cohorts of PLHIV were identified based on when patients first entered HIV care (a 2009 cohort and a 2010 cohort). The 2009 cohort was selected so that care practices during follow-up could be compared to care practices during initial enrollment.

For each clinic, patients were divided into groups based upon ART status (receiving ART or not receiving ART) and cohort (2009 cohort or 2010 cohort). Due to the large number of eligible patients, medical records were randomly selected for review using a random number generator. Each record within a group had an equal probability of being selected. The specific number of records selected from each of the groups was proportionate to the number of eligible patients in each of those groups. All individual medical records selected were available for review.

### Register review and medical record abstraction at HIV clinics

From HIV registers and medical records, data were collected on initiation of ART, CD4 count, ICF, TST eligibility, placement and interpretation of TSTs, and receipt of IPT. PLHIV with prior TB disease, prior positive TST, or prior IPT were ineligible for TST in accordance with national guidelines (7).

### Interviews of patients and staff

Staff from the 10 HIV clinics and six of nine TB clinics were interviewed to assess TB/HIV policies and practices; a convenience sample of staff was selected on the basis of availability and role at the clinic. A convenience sample of PLHIV attending HIV clinics was also interviewed to assess barriers and facilitators to returning for TST interpretation.

### Data analysis

Data abstracted from medical records were utilized to calculate the proportion of eligible patients receiving recommended TB/HIV care at TB clinics (e.g., receipt of CPT, receipt of ART, and known CD4 count results) and HIV clinics (e.g., TST for eligible PLHIV, IPT for PLHIV with a positive TST result). The chi-squared test was used to detect differences in proportions; results were considered to be significant if *P* < 0.05.

### Ethical review

The project received approval from the Guyana Ministry of Health Institutional Review Board (IRB). Additional review by the U.S. Centers for Disease Control and Prevention (CDC) IRB was not required because the CDC investigators for the study were determined not to be engaged in research as defined by U.S. government regulations. Consent was not obtained for medical record review, but abstracted information was anonymized and de-identified prior to analysis. For interviews, verbal informed consent was obtained from participating staff and patients as approved by the Guyana Ministry of Health IRB. Verbal consent was selected alternatively to written consent to address potential challenges with low literacy among participants. Verbal consent was documented by the interviewer on the anonymized data collection form assigned to each participant.

## RESULTS

### TB clinics

During the six-month study period in 2010, 461 cases of TB were diagnosed at the nine TB clinics. One clinic reported more than 75 TB cases, three clinics reported 25 to 75 cases, and five clinics reported fewer than 25 cases. HIV status was documented in the clinic register for 419 cases (90.9%) (Table 1), and 121 (28.9%) were HIV-infected. Medical records were reviewed for 63 of the 121 patients with TB/HIV; 56 (88.9%) received CPT, 49 (77.8%) had a documented CD4 count, and 33 (52.4%) received ART. Among the 45 PLHIV for whom dates of HIV diagnosis were available, 38 (84.4%) individuals knew their HIV status prior to TB diagnosis.

Among the 63 patients with TB/HIV, four (6.3%) were receiving CPT prior to TB diagnosis, 26 (41.3%) started CPT within two weeks of starting anti-TB treatment, 18 (28.6%) started CPT more than two weeks after starting anti-TB treatment, eight (12.7%) started CPT but did not have a documented date of CPT initiation, and seven (11.1%) did not receive CPT during anti-TB treatment. Eighteen (28.6%) had a documented CD4 count prior to TB diagnosis, 16 (25.4%) had a documented CD4 count within two weeks of starting anti-TB treatment, 13 (20.6%) had a documented CD4 count more than two weeks after starting anti-TB treatment, two (3.2%) had an unknown CD4 count date, and 14 (22.2%) had no documented CD4 count during anti-TB treatment.

Among the 35 PLHIV with a documented CD4 count ≤ 350, four (11.4%) were already receiving ART prior to TB diagnosis, two (5.7%) started ART within eight weeks of starting anti-TB treatment, 14 (40.0%) started ART at least eight weeks after starting anti-TB treatment, two (5.7%) started ART but had an unknown ART start date, and 13 (37.1%) did not receive ART during anti-TB treatment (Table 2). Among the 21 patients who knew their HIV status prior to TB diagnosis and had a known ART initiation date, eight (38.1%) were already receiving ART at the time of anti-TB treatment initiation.

**TABLE 1.** HIV diagnosis and management at tuberculosis (TB) clinics, Guyana, 2005-2006 and 2010

Indicator	2005-2006^a^(*n* = 253)		2010 (*n* = 461)	
	No.	%	No.	%
Persons for whom HIV status was established	194/253	76.7	419/461	90.9
Persons with HIV infection^b^	68/194	35.1	121/419	28.9
Knew HIV positive status before TB diagnosis^c^	57/68	83.8	38/45	84.4
Received cotrimoxazole preventive therapy	43/54	79.6	56/63	88.9
CD4 count done	38/54	70.4	49/63	77.8
Received antiretroviral therapy	18/54	33.3	33/63	52.4

***Source:*** TB clinic registers and medical records.

a Prepared by the authors from published data (4).

b According to register data; medical records were reviewed for a subset of patients reported to be HIV-infected.

c Defined as ≥ 28 days prior to anti-TB treatment start date; only 45/63 cases had documented dates of HIV diagnosis and anti-TB treatment start.

**TABLE 2.** Timing of antiretroviral therapy (ART) initiation among people with tuberculosis (TB)/HIV, by CD4 count^a^ at time of TB diagnosis, Guyana, 2010

Timing of initiation of ART^b^	CD4 count ≤ 350 (*n* = 35)		CD4 count > 350 (*n* = 14)	
	No.	%	No.	%
Prior to TB diagnosis^c^	4	11.4	2	14.3
0-8 weeks from anti-TB treatment start date	2	5.7	1	7.1
> 8 weeks from anti-TB treatment start date	14	40.0	3	21.4
Date unknown	2	5.7	1	7.1
ART not initiated	13	37.1	7	50.0

***Source:*** Medical records at TB clinics.

a Among patients with a known CD4 count.

b National guidelines for 2010 recommended initiation of ART within 8 weeks of TB diagnosis for CD4 count < 350; updated national guidelines now recommend initiation of ART at 2 weeks for CD4 count < 200, 2-4 weeks for CD4 count 200-500, and 4-8 weeks for CD4 count ≥ 500.

c Defined as ≥ 28 days prior to anti-TB treatment start date.

Among the 54 patients with TB/HIV and known anti-TB treatment outcomes, the following proportions had a favorable outcome: 0.0% (0 of 5) of those not receiving CPT or ART; 38.1% (8 of 21) of those receiving CPT only; and 77.8% (21 of 27) of those receiving both CPT and ART. One patient received ART only (and not CPT) and that patient had a favorable outcome; outcomes were missing for nine of 63 patients with TB/HIV (14.3%).

During interviews, three of seven TB clinic staff (42.9%) attributed delays in ART initiation to challenges in obtaining a CD4 count for persons newly diagnosed with TB/HIV and the three adherence counseling sessions required before initiating ART. Challenges in obtaining timely CD4 count results were cited by more remote sites because of delays in transporting specimens and receiving paper reports with CD4 cell count results.

### HIV clinics

Medical records were reviewed for 156 PLHIV receiving care at HIV clinics in 2010. ICF practices could not be assessed because of incomplete or discrepant documentation in the standard medical record used at HIV clinics (e.g., several medical records were marked as “no signs/symptoms of TB,” but had notations from the same visit of “cough” or “fever”).

**FIGURE 1:**
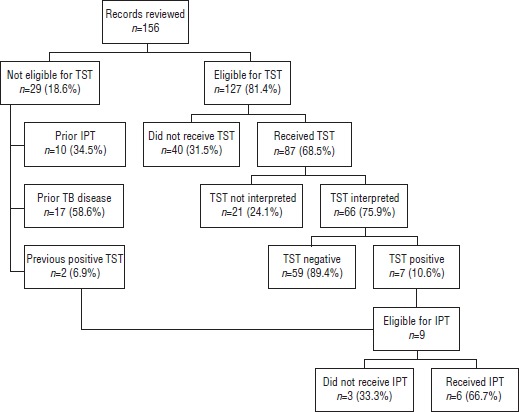
Flow diagram of the TST^a^ cascade in persons living with HIV; eligibility for TST (as per national guidelines); reasons for ineligibility; proportion with TST placed, of those eligible; proportion with TST interpreted, of those with TST placed; TST results, of those with TST interpreted; and proportion who received IPT,^b^ of those eligible, Guyana, 2010

***Source:*** Medical records at HIV clinics.

Overall, out of the cohort of 156 PLHIV, six (3.8%) received IPT. On the basis of Guyana’s 2010 national guidelines, 29 (18.6%) of the 156 patients were not eligible to receive a TST (Figure 1). Among the 127 remaining PLHIV eligible for TST, 87 (68.5%) received at least one TST in 2010. Of those 87 who received a TST, 66 (75.9%) had the test interpreted within the required 48–72 hours following placement, of which seven (10.6%) had a newly positive TST result. Of these seven, four (57.1%) received IPT. In addition, two patients with a previous positive TST result (and without prior IPT) both received IPT in 2010. In all, six of nine eligible individuals (66.7%) received IPT.

**TABLE 3.** Tuberculin skin testing (TST) and provision of isoniazid preventive therapy (IPT) for persons attending HIV clinics, Guyana, 2010

Indicator	2009 cohort^a^(*n* = 93)		2010 cohort^b^(*n* = 63)		P
	No.	%	No.	%	
Eligible for TST	74/93	79.6	53/63	84.1	0.47
Received TST	41/74	55.4	46/53	86.8	0.0002
Returned for TST interpretation	27/41	65.9	39/46	84.8	0.039
Positive TST result	4/27	14.8	3/39	7.7	NA^c^
Received IPT^d^	4/6	66.7	2/3	66.7	NA

***Source:*** Medical records at HIV clinics.

a Patients who first entered care at an HIV clinic in 2009 (or prior) and had at least one follow-up visit during July-December 2010.

b Patients who first entered care at an HIV clinic in 2010.

c NA: not applicable.

d Among the 2009 cohort, six persons were eligible for IPT: two patients with a previous positive TST and four patients with a newly positive TST. Both (two of two) patients with a previous positive TST received IPT; two of four patients with a newly positive TST received IPT.

Among the 156 PLHIV, eligibility for IPT and receipt of TST was stratified by cohort. Of the 93 patients in the 2009 cohort, 74 patients were eligible to receive a TST in 2010, 41 (55.4%) received a TST, and 27 (65.9%) returned for TST interpretation (Table 3). Of the 63 patients in the 2010 cohort, 53 were eligible to receive a TST, 46 (86.8%) received a TST, and 39 (84.8%) returned for TST interpretation.

A total of 37 staff at 10 HIV clinics were interviewed regarding TB/HIV practices. To facilitate routine ICF, staff suggested more explicit national guidelines and preprinted medical records with the WHO-recommended symptom review. Among 20 staff whose work responsibilities included placing or interpreting TSTs,15 (75.0%) stated that reminders in patient medical records or registers would make it easier to remember annual TST screening for eligible patients.

During interviews with 37 PLHIV who reported receiving a TST, the most frequent reasons for not returning for TST interpretation were wait times at the clinic (48.6%), getting time off from work (43.2%), and cost of travel (40.5%) (Table 4). PLHIV recommended the following facilitators to improve the percentage of patients returning for TST interpretation: education about why the patient needs to return (91.9%) and an explanation of when the patient needs to return (83.8%).

## DISCUSSION

Prompt diagnosis of HIV among patients with TB can lead to early initiation of life saving HIV care and treatment (5). The findings of this evaluation are consistent with previous research that established the reduced risk of death for people with TB/HIV receiving ART and CPT (8, 9); there was a gradient effect with more favorable anti-TB treatment outcomes for people with TB/HIV receiving ART and CPT when compared to CPT alone or no ART or CPT.

Comparing HIV-related services at TB clinics in 2005–2006 (4) and 2010, there were increases in the percentage of patients with known HIV status (76.7% to 90.9%), receipt of CPT (70.4% to 77.8%), and receipt of ART (33.3% to 52.4%) (Table 1). Although this study did not address possible causes for these increases, newly available onsite ART at TB clinics for patients with TB/HIV may have contributed to the observed improvement in receipt of ART.

Guyana’s national guidelines in 2010 recommended ART within eight weeks of anti-TB treatment initiation for PLHIV with a CD4 count ≤ 350 (6). However, this evaluation revealed that of those with a CD4 count ≤ 350 and not already on ART, only 5.7% initiated within eight weeks. In accordance with recent published studies (10–12) and WHO recommendations (5), Guyana’s updated national guidelines now recommend ART within eight weeks of starting anti-TB treatment for all patients with TB/HIV, regardless of CD4 count. However, due to concerns about immune reconstitution inflammatory syndrome, the recommended timing of ART initiation depends on CD4 count: two weeks for CD4 count < 200, two to four weeks for CD4 count 200–500, and four to eight weeks for CD4 count > 500 (13). In order to follow these recommendations, barriers to timely ART initiation (e.g., delays in obtaining CD4 count results and required ART adherence sessions) must be specifically addressed.

**TABLE 4.** Patient-identified barriers and facilitators to returning for tuberculin skin test interpretation, Guyana, December 2011

Barrier/facilitator	No.	%^a^
Barrier		
Long wait time at HIV clinic	18	48.6
Time off from work	16	43.2
Cost of travel to HIV clinic	15	40.5
Childcare or family duties	13	35.1
Not understanding reason for test	9	24.3
Not told of need to return	9	24.3
Inconvenient clinic hours	6	16.2
Not understanding need to return	6	16.2
Travel time to HIV clinic	6	16.2
Facilitator		
Staff explains why patient needs to return	34	91.9
Staff explains when patient needs to return	31	83.8
Skin test interpreted at closer site	25	67.6
Skin test interpreted at home	22	59.5
Skin test interpreted at work	16	43.2

***Source:*** Interviews with patients at HIV clinics (*n* = 37).

a Total percentage does not equal 100 because multiple choices may have been selected.

This study identified several areas for future development to reduce TB incidence among PLHIV in Guyana. First, ICF was incompletely or discrepantly documented at HIV clinics. Delayed evaluation or diagnosis of TB can have devastating consequences, both for individual patients and for infection control within an HIV clinic. Interviews with providers suggested that more explicit national guidelines as well as practical reminders in HIV clinics would be helpful to ensure that ICF is implemented as a routine practice at each visit. On the basis of these findings, the standard chart medical record used at HIV clinics has been revised to better reflect the WHO-recommended symptom review and make it easier for providers to document findings.

Second, these results showed that 84.4% of patients with TB/HIV in 2010 knew their HIV status prior to TB diagnosis and yet only 38.1% were receiving ART. This finding may be partly explained by a sixfold increase in HIV testing among the general population in Guyana from 2005 to 2010 (14). In addition, national indicators demonstrate that only 27.4% of persons who know their HIV-positive status attended HIV clinics in 2010 (although 72.6% of those attending HIV clinics received ART) (14). Therefore, future efforts to increase enrollment of patients in HIV care, routine implementation of the Three I’s, and early initiation of ART (5, 15, 16) could have a sizeable impact on reducing TB incidence among PLHIV in Guyana.

Third, the results of this evaluation show that only 3.8% of all PLHIV receiving care at HIV clinics were receiving IPT. The number of PLHIV receiving IPT is the standard indicator utilized by WHO when assessing TB/HIV activities (1). However, this value alone does not reveal the reasons for low rates of IPT in a given country, nor does it help inform potential strategies for scale-up. This evaluation demonstrated that, in Guyana, placement of annual follow-up TSTs was the most important barrier to provision of IPT; only 55.4% of eligible PLHIV in follow-up care received a TST in 2010 (compared to 86.8% among PLHIV first entering care). This disparity signals an important area for future scaleup, as annual follow-up TSTs can identify two high-risk groups that might benefit from IPT: 1) PLHIV who are severely immunocompromised and might have anergic TST reactions at enrollment and 2) PLHIV who are infected with TB after enrollment. To achieve this goal, additional emphasis should be placed on annual TST placement, with chart and clinic reminders as well as educational programs for providers.

One of the challenges of routine TST implementation is the need for test interpretation within 36–48 hours following placement, usually requiring a return visit by the patient to the clinic. As a result, using interferon gamma release assays (IGRAs) in place of TSTs could eliminate the need for a return visit. However, IGRA implementation would likely be challenging in Guyana, given the higher cost of the test (in comparison to TST) and the laboratory capacity requirements for incubating, transporting, and analyzing specimens (17). In addition, overall rates of return for TST interpretation were reasonably high (75.8%), and low rates of annual TST placement (55.4%) represented the more important barrier to IPT. Therefore, even if IGRAs were adopted, efforts to improve annual testing would still be necessary to improve scale-up of IPT.

Based upon low rates of IPT uptake worldwide, WHO guidelines state that “TST is not a requirement for initiating IPT in people living with HIV,” but that “TST can be used when feasible” (5). Although only 3.8% of PLHIV in this study received IPT, the results of this evaluation do not support a policy of universal IPT in Guyana. Previous studies have established that PLHIV with a positive TST result will benefit most from IPT (18). Given the relatively low prevalence of a positive TST result in this study (10.6%), a policy of universal IPT would result in unnecessary treatment of nine out of 10 PLHIV (19–21). In addition, despite low rates of annual TST placement, most patients did return for interpretation, and most of those with positive TST results received IPT. Finally, Guyana has decades of experience in training health care workers to place and interpret TSTs and in decentralizing tuberculin reagents. Given these factors, targeted IPT is a feasible and efficient strategy to reduce TB incidence among PLHIV in Guyana.

### Limitations

This evaluation had several limitations. First, although the clinics in this assessment provided care for most persons with TB or HIV in Guyana (> 90% of all TB cases and > 80% of all persons attending HIV clinics), these results may not be applicable to smaller, remote clinics in Guyana not included in this evaluation. Some of these more remote clinics may face unique challenges, such as transportation for patients to clinic sites and timeliness of CD4 count results. Three additional TB clinics beyond those evaluated in 2006 were examined in the 2010 assessment in an effort to include more remote sites.

Another limitation was that staff and patient interviews were conducted among a convenience sample and therefore should not be considered representative. However, the findings of these interviews were useful to contextualize the results from the review of clinic medical records, particularly by identifying potential barriers to best practices.

A final limitation was the small number of patients with a newly positive TST in the study cohort; observations of high initiation rates of IPT may not be generalizable, and ongoing assessments including larger numbers of patients with a positive TST result will be essential to ensure successful IPT scale-up.

### Conclusions

Between 2005 and 2010, Guyana made substantial progress in increasing HIV testing for people with TB, and HIV-related care for people with TB/HIV, but areas for improvement remain. Specifically, continued scaleup of ART is needed for patients with TB/HIV. Use of TST to focus IPT for PLHIV is feasible and efficient, and improving rates of annual TST screening will allow for further expansion of IPT.

### Acknowledgments.

The following individuals provided invaluable assistance with data collection: Leta DeJonge, Diana Dhanraj, Emma Johns, and Candice Kwan. The authors greatly appreciate the contributions of Maxia Dong, Andrea Lambert, Vivienne Lowe, and Mallika Mootoo. They would also like to thank the patients and staff at study clinics who participated in interviews.

### Disclaimers.

Authors hold sole responsibility for the views expressed in the manuscript, which may not necessarily reflect the opinion or policy of the *RPSP/PAJPH* or the Pan American Health Organization (PAHO). The findings in this report are those of the authors and do not necessarily represent the official position of the U.S. Centers for Disease Control and Prevention.

## Footnotes

^a^**TST:** tuberculin skin test.

^b^**IPT:** isoniazid preventive therapy
